# Psychopathological and psychosocial factors influencing physical health of people with mental disorders

**DOI:** 10.1192/j.eurpsy.2021.402

**Published:** 2021-08-13

**Authors:** F. Zinno, C. Palummo, L. Giannelli, A. Pitocco, A. Carello, E. Barone, V. Giallonardo, G. Sampogna, M. Luciano, V. Del Vecchio, A. Fiorillo

**Affiliations:** 1 Department Of Psychiatry, University of Campania “Luigi Vanvitelli”, Naples, Italy; 2 Department Of Pschiatry, University of Campania “Luigi Vanvitelli”, Naples, Italy; 3 Department Of Psychiatry, University of Campania “Luigi Vanvitelli”, Napoli, Italy; 4 Department Of Psychiatry, University of Campania, Naples, Italy

**Keywords:** Stigma, Mental disorders, Physical health, chronic diseases

## Abstract

**Introduction:**

Severe mental disorders (SMD) are associated with higher morbidity rates and poorer health outcomes compared to the general population. They are more likely to be overweight, to be affected by cardiovascular diseases, and to have higher risk factors for chronic diseases.

**Objectives:**

To assess physical health in a sample of patients with SMD and to investigate which mental health-related factors and other psychosocial outcomes could be considered predictors of poor physical health.

**Methods:**

Patients referring to the psychiatric outpatients unit of the University of Campania “L. Vanvitelli” were recruited, and were assessed through validated assessment instruments exploring psychopathological status, global functioning and stigma. Physical health was assessed with an ad-hoc anthropometric schedule. A blood sample has been collected to assess levels of cholesterol, blood glucose, triglycerides, and blood insulin.

**Results:**

75 patients have been recruited, with a mean age of 45.63±11.84 years. 30% of the sample had a diagnosis of psychosis, 27% of depression and 43% of bipolar disorder. A higher BMI is predicted by higher number of hospitalizations, a reduced score at MANSA (p<.000), and PSP (p<.05), and higher score at ISMI and BPRS (p<.05). A higher cardiovascular risk is predicted by a reduced MANSA score (p<.000), a higher ISMI score and a poorer adherence to pharmacological treatments (p<.05). Higher ISMI score (p<.0001) and number of hospitalizations (p<.05) are predictors of insulin-resistance.

**Conclusions:**

Our study shows that psychosocial domains negatively influence physical health outcome. It is necessary to disseminate an integrated psychosocial intervention in order to improve patients’ physical health.

**Disclosure:**

No significant relationships.

